# MSC-Derived Exosomes-Based Therapy for Peripheral Nerve Injury: A Novel Therapeutic Strategy

**DOI:** 10.1155/2019/6458237

**Published:** 2019-08-18

**Authors:** Ruiqi Dong, Yumei Liu, Yuxiang Yang, Haojie Wang, Yaolu Xu, Ziqiang Zhang

**Affiliations:** College of Animal Science and Technology, Henan University of Science and Technology, Luoyang, Henan 471023, China

## Abstract

Although significant advances have been made in synthetic nerve conduits and surgical techniques, complete regeneration following peripheral nerve injury (PNI) remains far from optimized. The repair of PNI is a highly heterogeneous process involving changes in Schwann cell phenotypes, the activation of macrophages, and the reconstruction of the vascular network. At present, the efficacy of MSC-based therapeutic strategies for PNI can be attributed to paracrine secretion. Exosomes, as a product of paracrine secretion, are considered to be an important regulatory mediator. Furthermore, accumulating evidence has demonstrated that exosomes from mesenchymal stem cells (MSCs) can shuttle bioactive components (proteins, lipids, mRNA, miRNA, lncRNA, circRNA, and DNA) that participate in almost all of the abovementioned processes. Thus, MSC exosomes may represent a novel therapeutic tool for PNI. In this review, we discuss the current understanding of MSC exosomes related to peripheral nerve repair and provide insights for developing a cell-free MSC therapeutic strategy for PNI.

## 1. Introduction

Peripheral nerve injury (PNI) is a common neurological disease that seriously threatens human health [1]. In general, it occurs in 2.8%-5% of polytrauma patients, may result in sensory and motor dysfunction, and may even cause permanent disability [2, 3]. Axonal outgrowth following PNI is orchestrated by many factors, such as the transformation of the phenotype of Schwann cells (SCs), the infiltration of immune cells, and neurovascular regeneration [4–6]. Regenerating axons grow only 1 mm per day, which makes the regeneration of peripheral nerves a challenge [7, 8]. Furthermore, the prolonged denervation of proximal nerves may increase the likelihood of irreversible atrophy of the innervated organs [9, 10]. Additionally, the loss of nerve tissue associated with PNI also poses additional problems for nerve regeneration [11]. Although autologous nerve graft is the gold standard technique, the use of nerve grafts is limited due to the shortage of donor sources and the possible loss of neurological function in the donor site [12, 13]. Over the past decade, various studies have focused on developing new methods to promote axonal regeneration without sacrificing other healthy functioning nerves [14, 15]. Although some synthetic nerve conduits permeated with an abundance of SCs or stem cells have been demonstrated to be beneficial, the outcomes of PNI remain far from ideal [16–18].

Mesenchymal stem cell (MSC)-based therapy is thought to be a promising strategy for PNI [19–21]. Previous studies have shown that transplanted MSCs can differentiate into SCs (an important type of glial cell in the peripheral nerve system that mediates axonal regeneration) in vivo, providing the support and nutrition needed for axonal growth [22, 23]. The mechanism by which MSCs facilitate nerve repair has not yet been clarified. Previous studies have shown that MSCs can adopt a Schwann cell phenotype in vitro after they are cocultured with peripheral nerve extracts from the distal segment of a damaged sciatic nerve [24, 25]. However, Sowa et al. found that transplanted MSCs significantly promote axonal outgrowth, the formation of myelin, and the recovery of denervated muscle atrophy; however, transplanted MSCs do not differentiate into Schwann cells [26]. This indicates that the therapeutic effect of transplanted MSCs is more likely to be attributed to the indirect regeneration of endogenous SCs through a cellular paracrine mechanism than through transdifferentiation. 

Exosomes are membrane nanovesicles that are found in almost all biological fluids, such as blood, urine, breast milk, ascites, and saliva [27, 28]. As important mediators of paracrine mechanisms, they have begun to be noticed. Recent studies have indicated that exosomes are associated with pathological and physiological conditions, such as neurodegenerative diseases [29–31], tumors [32–35], and tissue fibrosis [36–41]. Exosomes contain cellular signaling molecules (proteins, lipids, mRNA, miRNA, lncRNA, circRNA, and DNA) that mediate intercellular communication by horizontally transferring these types of cargo [42, 43]. Emerging evidence has shown that exosomes derived from MSCs exert beneficial effects on a variety of disease models, including models of myocardial infarction [44–47], hepatic fibrosis [48–50], and even cancer [51–56]. Significantly, MSC-derived exosomes have the potential to be used for therapeutic strategies for peripheral nerve regeneration. Thus, this review summarizes current studies on MSC exosomes-based therapeutic strategies and discusses the future implications of MSC exosomes as an agent for treating peripheral nerve injury.

## 2. Concise Review: Current Knowledge Regarding the Use of MSCs for Tissue Repair

MSCs are a population of undifferentiated adult stem cells that possess self-renewal capability, low immunogenicity, and multilineage differentiation potential [57]. As a preclinical agent, MSCs have achieved exciting outcomes in the field of tissue regeneration since they were first described in 1968. Autologous or allogenic MSC transplantation can remarkably improve clinical outcomes via mediating the inflammatory response, modulating cell apoptosis, and promoting cell proliferation [58]. As of now, many studies on MSC-based therapy are ongoing or have been completed, and it is generally considered to be effective and safe.

The clinical testing of and research on MSCs for extensively repairing tissue injury is predicated on the hypothesis that transplanted MSCs can transdifferentiate into appropriate cell types and migrate to target tissues to replace injured cells after transplantation. An increasing number of studies, however, have shown that the number of these cells engrafted within target tissues is extremely low (<1% of cells) [59]. Most transplanted MSCs are found to be trapped in the liver and spleen. Whether engrafted MSCs differentiate into the desired cells in vivo is also controversial. In addition, there are some inherent disadvantages of MSC-based therapy, including the instability of the cellular phenotype, high cost, issues of cellular origin and transport before transplantation, microinfarction caused by MSCs lodged in the pulmonary microvasculature, and ethical issues [60–64]. Therefore, a novel cell-free therapy for PNI with a similar efficacy as that of MSCs needs to be further developed. Notably, MSC-conditioned medium containing MSC secretomes can replicate the therapeutic effects of MSCs [65, 66]. For decades, many investigators have worked to find active secretome of MSCs to explain the mechanism of therapy. Although these active factors are considered promising, there is no single factor that fully explains the efficacy of MSCs. In 2010, Lai et al. first demonstrated that exosomes derived from MSCs can alleviate myocardial ischemia/reperfusion injury [67]. Subsequent studies have also demonstrated that the therapeutic effects of MSCs are closely associated with exosomes secreted by MSCs. MSC exosomes seem to exert comparable effects as those of their parental cells. These novel findings provide new insight into the repair of injured tissue by MSCs and intercellular communication between MSCs and recipient cells and offer a new approach for the development of biological agents for tissue repair. Therefore, MSC exosomes may become a novel therapeutic target.

## 3. Biogenesis and Biocharacteristic of Exosomes

EVs are membrane-contained vesicles secreted by almost all cell types, such as immunocytes, MSCs, tumor cells, Schwann cells, and epithelial cells, into the extracellular space [68]. It has been demonstrated that EVs exhibit diverse intercellular communication biofunctions, such as antigen presentation, tumor progression and metastasis, and tissue regeneration regulation [69–71]. Exosomes, an important subclass of EVs, have gained much attention in biomedical research.

According to the origin and biogenesis of EVs, EVs can be divided into two main types, namely, exosomes and microvesicles (MVs) [72]. Exosomes are formed by inward budding inside intracellular endosomes, which leads to the formation of multivesicular bodies (MVBs) that then fuse with the PM and are released outside the internal vesicles, while other EVs, such as MVs, are budding vesicles that are shed directly from the PM [68]. Furthermore, exosomes can be distinguished from other EVs by their size. Exosomes are nanosized membrane vesicles that are 40-150 nm in diameter, whereas MVs range from approximately 100 to 1000 nm. Obviously, exosomes are narrower than MVs in the diameter. The characteristics of exosomes and MVs are summarized in Table 1. Upon the observation of exosomes by transmission electron microscopy, their morphology was described as “cup-shaped” [73]. Exosomes are endocytic membrane-derived vesicles with a bilayer lipid structure that consists of cholesterol, ceramide, phospholipids, and glycerophospholipids with long and saturated fatty-acyl chains [74, 75]. The bilayer membrane provides a protected and controlled internal microenvironment for the contents, ensuring that the cargo is not degraded and that it can be moved over long distances [76]. In addition, due to the low number of membrane-bound proteins, such as major histocompatibility complex molecules, in exosomes, they are less immunogenic than their parental cells. This feature can allow them to overcome immune rejection and avoid being cleared by immunocytes [77, 78].

**Figure 1:**
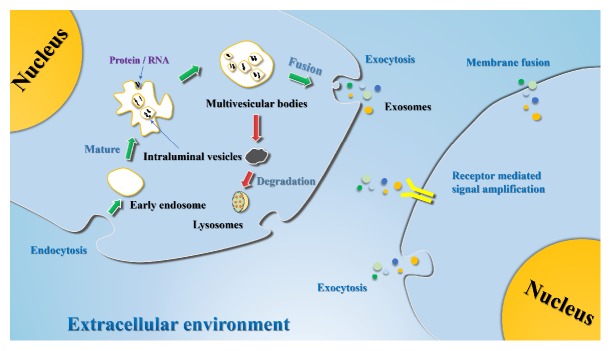
Biogenesis and uptake of exosomes. Endosomes are formed by an invagination in the plasma membrane of cells. As endosomes mature, the endosomal membrane constantly buds inward to form multivesicular bodies (MVBs). Exosomes are released through the fusion of multivesicular bodies with the plasma membrane. Once an exosome is released, receptor cells uptake the exosome through endocytosis, membrane fusion, or receptor-mediated internalization.

The secretion of exosomes involves a complicated endocytic pathway. At the beginning of the process, endosomes invaginate from a special plasma membrane region into the cytoplasm. As endosomes mature, the endosomal membrane constantly buds inward to form intraluminal vesicles (IVs). At this stage, the endosomes are called multivesicular bodies (MVBs). After the MVBs fuse with the cell membrane, the MVBs are released into the extracellular space. The released vesicles are called exosomes. At the same time, some MVBs are transferred to lysosomes for degradation [79]. Interestingly, in the process of IV and MVB biogenesis, there are two distinct molecular mechanisms, namely, endosomal sorting complex required for transport (ESCRT)-dependent and ESCRT-independent mechanisms [80, 81]. These are thought to be complex molecular mechanisms that sort and encapsulate specific molecules into IVs. When exosomes are released into the extracellular environment, they can adhere to the surface of recipient cells, interact with lipid ligand receptors, and then be internalized by endocytic uptake or fusion with the cell membrane [79] (see Figure 1).

Compared with other EVs, exosomes also possess their own expression profiles. Exosomes contain abundant proteins associated with membrane transport and fusion, including annexins, ESCRT and Rab GTPases, actin, and *β*-tubulin. Exosomes are also enriched with specific protein markers, such as tetraspanins (CD9, CD63, CD81, CD82), heat shock proteins (HSP60, HSP70, HSP90), tumor susceptibility gene 101 (TSG101), and ALG-2-interacting protein X (ALIX) [82]. Thus, enzyme-linked immunosorbent assay (ELISA), flow cytometry, and Western blotting are frequently used to detect exosomal biomarkers by analyzing specific proteins [83, 84]. It has been reported that exosomes carry mRNA, ribosomal RNA (rRNA), circRNAs DNA, long noncoding RNA (lncRNA), and some cytokines [85]. Although exosomal miRNAs only account for a small fraction of loaded molecules, many studies have indicated that exosomes are thought to be an important mediator responsible for regulating the phenotype and physiological state of recipient cells by horizontally transferring miRNAs [86]. Mechanically, miRNAs can bind to targeted mRNAs, resulting in mRNA decay or translation dampening and ultimately the regulation of expression [87]. Although their cytoskeleton and cellular metabolism are the same, their cargo and composition are different and depend on the cellular origin and different physiological and pathological states of cells [88]. Obviously, exosomes can also encapsulate loaded molecules based on different functional requirements and external stimuli. In other words, the contents carried by exosomes can be shuttled to regulate cell biological behavior.

## 4. MSC Exosomes Mediate Axonal Outgrowth

It is well known that endogenous SCs contribute mainly to axonal development, maturation, and regeneration. SCs play a central role in maintaining the homeostasis of the peripheral nervous system and promoting regeneration following injury [89]. Recent studies have indicated that exosomes derived from SCs may be a critical mediator of communication with axons [90]. In other words, a number of protein and genetic components are transferred from SCs to axons though exosomes, thereby promoting axonal regeneration. A study from 2013 showed that exosomes from SCs can be specifically internalized by axons. It was subsequently observed that exosomes from SCs significantly increase axonal regeneration in vitro and enhance regeneration after sciatic nerve injury in vivo [91]. Mechanically, exosomes shift growth cone morphology to a proregenerative phenotype and decrease the activity of the GTPase RhoA, which is involved in growth cone collapse and axon retraction [91, 92]. The above studies suggest that exosomes from glial cells may be a promising therapeutic strategy for facilitating axonal outgrowth. However, harvesting exosomes from SCs requires sacrificing normal neural tissue [93]. More importantly, SCs are difficult to culture in vitro since they are terminal cells. The above shortcoming is the main challenge for SC-based therapeutic strategies for repairing nerve injury. Therefore, subsequent research is needed to find an alternative cell type with efficiency similar to that of SCs.

Fortunately, previous studies have demonstrated that MSCs, similar to SCs, exert a beneficial effect by promoting peripheral nerve regeneration. Many studies have indicated that MSC-derived exosomes, as cellular paracrine products, may play a major role in recovery after PNI. In 2016, Lopez-Verrilli et al. reported that MSC exosomes from different sources, including menstrual MSCs and bone marrow mesenchymal stem cells (BMMSCs), can facilitate the neurite outgrowth of dorsal root ganglia neurons and cortical neurons [94]. Another study demonstrated that neuroprotection conferred by MSC exosomes (the source of adipose-derived stem cells) is attributed to the activation of the PI3K/AKT signaling pathway [95]. Furthermore, a recent study reported that gingiva-derived mesenchymal stem cell (GMSC)-derived EVs can induce the upregulation of SC dedifferentiation/repair phenotype-related genes by activating the c-JUN-governed repair phenotype of SCs, thereby facilitating peripheral nerve regeneration [96]. Overall, increasing evidence has demonstrated that MSC exosomes have the potential to be a promising mediator of PNI regeneration.

### 4.1. MSC Exosomes Mediate the Intercellular Transport of miRNAs to Induce Axonal Outgrowth

Studies have indicated that the miRNA expression profile of SCs and neuron cell bodies following PNI is abnormal, which suggests that local genetic effects largely mediate regeneration after peripheral nerve injury [97, 98]. Moreover, these specifically expressed miRNAs are involved in the proliferation and phenotypic switch of SCs, neurogenesis, axonal outgrowth, and macrophage migration. For example, the overexpression of specific miRNAs (miR-23a, miR-200, miR-133b, miR-17-92 cluster) can promote neurogenesis and myelination [99–102]. Previous studies have reported that miRNAs are transferred from neuronal cell bodies to axons, while recent studies have demonstrated that miRNAs called exosomal shuttle RNAs can be transferred between cells though exosomes [103, 104]. This discovery suggests that exosomes can be packaged with beneficial miRNAs to shuttle these genetic components, thereby mediating neural plasticity and axonal outgrowth.

Emerging studies have shown that MSC exosomes can mediate cell-cell communication by transferring miRNAs to recipient neurons to promote axonal growth. Mead et al. found that BMMSC-derived exosomes can significantly promote the survival of retinal ganglion cells and axonal regeneration. The neuroprotective effect is attenuated by the knockdown of Argonaute 2, a key miRNA effector molecule, confirming that the effect is attributed to the delivery of exosomal miRNAs [105]. Xin et al. demonstrated that, compared with native MSC exosomes, tailored exosomes enriched with the miR-17-92 cluster can enhance neurological recovery following stroke by activating the PI3K/protein kinase B/mechanistic target of rapamycin/glycogen synthase kinase 3*β* signaling pathway [106]. In addition, tailored exosomes enriched with the miR-17-92 cluster can also activate the PTEN/mTOR signaling pathway and enhance axonal growth by delivering miRNA cargo in vitro [107]. Recently, a study showed that MSC exosomes enriched with miR-133b further stimulate the release of exosomes from astrocytes, indirectly enhancing neural plasticity and neurite outgrowth [108]. MSC exosomes may target neural cells in the neurogenic niche by transmitting exosomal miRNAs to contribute to neurite outgrowth. This may prove to be a meaningful finding for the treatment of PNI.

### 4.2. MSC Exosomes Shuttle Neurotrophic Factors to Promote Axonal Regeneration

In addition to transmitting genetic components, MSC exosomes contain numerous neurotrophic factors that also play an important role in axonal regeneration. To some extent, the components of MSC exosomes depend on the culture and physiological conditions of the original cells, which influence the biological function of MSC exosomes. A study indicated that exosomes from PEDF-modified ADSCs can activate autophagy and suppress neuronal apoptosis, thereby ameliorating cerebral I/R injury [109]. This study suggests that modifying MSCs to increase the level of bioactive molecular cargo may be a promising approach for accelerating recovery from PNI. Recently, Bucan et al. noted that MSC exosomes can be internalized by SCs in vitro, significantly increasing the proliferation of SCs. In vivo, MSC exosomes are selectively internalized by axons to promote axonal regeneration [110]. Although this study did not indicate the specific mechanism of peripheral nerve regeneration, it was the first to demonstrate that MSC exosomes are enriched with multiple neurotrophic factors, such as glial cell-derived neurotrophic factor (GDNF), fibroblast growth factor-1 (FGF-1), brain-derived neurotrophic factor (BDNF), insulin-like growth factor-1 (IGF-1), and nerve growth factor (NGF). The beneficial effects exerted by MSC exosomes are closely related to shuttling these neurotrophic factors. Thus, the presence of bioactive molecules related to nerve repair in MSC exosomes provides the possibility of regulating recovery from denervation muscle atrophy, neural survival, and axonal outgrowth.

## 5. MSC Exosomes Modulate Neuroinflammation in Peripheral Nerve Regeneration

Previous studies have shown that axonal regeneration after PNI is not completely mediated by SCs but rather by macrophages. Although the cellular immune response to nerve injury and infection is relatively conserved in the PNS, a majority of immune cells, such as phagocytic neutrophils and macrophages, migrate to the damaged site within hours or days after peripheral nerve injury [111]. Neuroinflammation plays a crucial role in recovery from PNI. Once a nerve is damaged, myelinating Schwann cells that undergo dedifferentiation are primarily activated in the distal portion of the nerve. Dedifferentiated Schwann cells begin to clear injured myelin and cell debris in a process known as Wallerian degeneration (WD). The release of chemokines and proinflammatory cytokines by dedifferentiated Schwann cells leads to the promotion of the neuroinflammatory response [112, 113]. Neuroinflammatory events lead to the recruitment of circulating macrophages and other peripheral immune cells to the injury site. Circulating macrophages and resident macrophages further facilitate the clearance of myelin and axonal debris, which is necessary for the regeneration of axons because molecules from degenerated axons exert an inhibitory effect on axonal growth, in later stages of WD [114]. Macrophages and other phagocytes accumulate in the region of the injured neuronal cell body in addition to the distal stump. These cells trigger a conditioning lesion response, which is the process by which neurons promote regeneration [115]. However, neuroinflammation exerts a double-edged sword effect. Although neuroinflammation has several beneficial effects in the process of recovery from PNI, it is crucial to nerve regeneration for this inflammatory response to be shut down. Excessive inflammatory responses not only hinder nerve regeneration but also are closely related to neuropathic pain. Hence, promoting a microenvironment that allows a suitable level of inflammation and regeneration might be a crucial target for PNI.

### 5.1. Immunomodulatory Characteristics of MSC Exosomes

It is well known that MSCs possess immunomodulatory properties though secreting various soluble immunomodulatory mediators, including transforming growth factor (TGF-*β*), interferon-*γ* (IFN-*γ*), indoleamine 2,3-dioxygenase (IDO), prostaglandin E_2_ (PGE_2_), and heme oxygenase-1, interleukin-10 (IL-10) [116]. However, studies have shown that defective IFN-*γ* receptors in MSCs and IDO inhibitors induce the loss of IFN-*γ* and IDO, which does not completely compromise the immune activity of MSCs [117–119]. Hence, the immunomodulatory activities of MSCs cannot be sufficiently explained by the release of the above-mentioned single soluble mediators but is mediated synergistically by multiple complex factors. Among various paracrine factors, MSC-derived exosomes are considered to be major immunomodulatory mediators that contain >200 immunomodulatory proteins [120]. Indeed, many studies have shown that MSC exosomes exert positive immunomodulatory effects in models of different pathologic conditions. In 2014, Zhang and his colleague noted that the treatment of THP-1 monocytes with MSC exosomes can induce high levels of anti-inflammatory IL10 and TGF-*β*1 in vitro and also attenuate the expression of proinflammatory IL1B, IL6, TNFA, and IL12P40. Additionally, they observed that MSC exosomes induce regulatory T cells (Tregs), which are recognized as a specialized subset of CD4^+^ T cells that play a role in the establishment and maintenance of immune tolerance, to enhance the survival of allogenic skin grafts [121]. Recently, a study reported that MSC exosomes mediate multifaceted cellular processes, including migration, proliferation, matrix synthesis, macrophage infiltration, and cytokine production, to promote tissues repair. The investigator attributed the positive effect to the presence of CD73, which induces the phosphorylation of both AKT and ERK, thereby activating AKT/ERK signaling, in MSC exosomes [122]. In brief, these data suggest that the diverse factors expressed by MSC exosomes have an immunomodulatory effect rather than an immunosuppressive effect, which makes MSC exosomes a promising candidate for immunomodulation after PNI.

### 5.2. MSC Exosomes Mediate the Phenotypic Transformation of Macrophages to Promote Axonal Regeneration

Recent studies have indicated that MSC exosomes can regulate the plasticity of macrophages to facilitate polarization into anti-inflammatory phenotypes, thereby reducing the production of proinflammatory cytokines.

Compared with unconditioned MSCs, LPS-preconditioned MSCs not only enhance the paracrine effect to increase the production of exosomes but also increase the accumulation of miRNAs associated with immunomodulation in MSC exosomes. LPS-preconditioned MSC-derived exosomes (LPS pre-Exo) can upregulate anti-inflammatory cytokines by promoting M2 macrophage activation. Mechanically, LPS pre-Exo mediate the TLR 4/NF-*κ*B/STAT-3/AKT regulatory signaling pathway, which regulates macrophage plasticity by shuttling let-7b to alleviate inflammation and maintaining intercellular homeostasis [123]. In addition, MSC exosomes also have the potential to improve brain plasticity by resolving neuroinflammation. Microglial, which are brain-resident immune cells that play a key role in homeostasis under normal conditions, are equivalent to macrophages in the central nervous system (CNS). Recently, it was confirmed that MSC exosomes can reverse neuronal damage by transmitting exosomal miR-30d-5p to inhibit the autophagy of microglia, ultimately promoting the polarization of microglia to an anti-inflammatory phenotype [124]. This study not only explained the mechanisms by which MSC exosomes mediate neuroinflammation to promote neurogenesis but also suggested that MSC exosomes may be a promising vehicle for gene delivery to attenuate neuroinflammation.

The neuroinflammation triggered by PNI is complex, and the entire reaction is precisely orchestrated and involves multiple immune cells and regulating factors [125]. For decades, the polarization of macrophages post-PNI has been controversial. Ydens et al. observed that only macrophages of the M2 phenotype are present following nerve axotomy, while another investigator reported that macrophages acquire the M1 phenotype post-PNI [126]. This difference in observation may be attributed to differences in time points, the injury model, and macrophage markers used. Furthermore, due to the presence of various stimuli in vivo, the exposure of macrophages to such environments may result in a complex mixed phenotype population. Collectively, although there are few reports that MSC exosomes mediate neuroinflammation to promote axonal outgrowth, this may be a research target for the future.

## 6. MSC Exosomes Mediate Vascular Regeneration in Peripheral Nerve Regeneration

The reconstruction of the vascular network provides a regenerative microenvironment for the facilitation of axonal growth during peripheral nerve repair [127]. Therefore, maintaining vascular integrity following PNI may be another target for the treatment of peripheral nerve regeneration. Recently, MSC-derived exosomes have attracted attention as paracrine promoters of angiogenesis and are thought to be valuable therapeutic tools for vascular remodeling. Exosomes from induced pluripotent stem cell-derived mesenchymal stem cells promote local angiogenesis by activating the PI3K/AKT signaling pathway in endothelial cells [128]. Additionally, Gong et al. showed that proangiogenic miRNAs can be transferred within endothelial cells though MSCs generated exosomes to improve vascular plasticity [129].

Interestingly, some studies have shown that MSC-derived exosomes can induce angiogenesis, thereby decreasing neurologic deficits. In 2013, Xin et al. demonstrated that the intravenous administration of MSC-generated exosomes can improve functional recovery and enhance neurite remodeling, neurogenesis, and angiogenesis [130]. Similar effects were confirmed in another study in which MSC-generated exosomes were shown to promote endogenous angiogenesis and neurogenesis and reduce inflammation in rats [131]. In summary, the above studies indicate that exosomes derived from MSCs may be mediators of communication with vascular endothelial cells to improve the plasticity of blood vessels after nerve injury. MSC exosomes may provide a new opportunity for PNI repair by facilitating neurovascular regeneration. However, there have been few reports that MSC exosomes enhance angiogenesis and promote peripheral nerve regeneration. Therefore, future studies are warranted to more carefully determine whether MSC exosomes can promote neurovascular regeneration in the process of peripheral nerve repair.

## 7. MSC Exosomes: Advantages and Challenges for Recovery from PNI

Recent studies have demonstrated that exosomes from MSCs play an important role in nerve regeneration following injury (see Table 2). In the future, MSC exosome-based therapy could be considered a potential alterative therapy for PNI. More attention should be paid to the safety, challenges, and risks of MSC exosomes as an emerging therapeutic agent, as these factors could provide a reasonable basis for the application of MSC exosomes for PNI repair. Therefore, a reasonable explanation of the advantages and challenges of MSC exosomes is required.

It is widely recognized that MSC-based therapy has some inherent risks, including tumor formation, immune rejection, microcirculatory obstruction, and arrhythmia. Unlike MSCs, MSC exosomes are a type of nonliving cell vesicle, and thus their use could radically eliminate the risk of MSC transplantation [132]. Exosomes possess lower immunogenicity because they contain fewer membrane-bound proteins compared with their parental cells [133]. Since MSC exosomes have nanoscale structures and because of the composition of their membranes and the adhesive proteins embedded within them, they can cross biological barriers [91–93]. Furthermore, the bilayer lipid structure of their membrane promotes the stability and bioavailability of their cargo. These features may make them promising natural nanocarriers for transporting drugs, genetic materials, proteins, and lipids and regulating the biological functions of target cells. Moreover, MSC exosomes are also easier to store and can be stored without potentially toxic cryopreservatives in long term at -20°C, greatly reducing the potential for toxic side effects of chemical agents [134]. Unlike those of stem cells, the vitality and potency of MSC exosomes need not be monitored and maintained during manufacturing and storage.

Despite these advantages of the use of MSC exosomes for treating PNI, the challenges cannot be ignored. Currently, one of the major challenges is that there is no standardized technique for the isolation, quantification, and purification of MSC exosomes. In addition, there is no guidance, supervision, or ethical safety assessment. It is necessary to determine the effective route of administration for PNI repair. It is worth exploring whether MSC exosomes are effective when administered systemically, such as through intravenous, subcuticular, or intramuscular routes, which are used for traditional drug therapy. In the last decade, most studies have tended to focus on the use of MSCs combined with artificial neural conduits to generate nerve grafts that mimic autologous nerves for repairing PNI. This approach also gives us great insight into the use of neural conduits combined with MSC exosomes to promote peripheral nerve regeneration. However, there have been few studies that have reported that the implantation of a graft conduit bathed with MSC exosomes promotes the repair of PNI. In addition, although many studies have observed that MSC exosomes can accelerate peripheral nerve regeneration after injury, the molecular mechanisms underlying nerve regeneration have not yet been elucidated. Hence, much work is needed to overcome the abovementioned risks and challenges to allow the translation of MSC exosomes as clinical agents for PNI in the near future.

## 8. Conclusion and Perspectives for the Future

Presently, MSC exosomes are widely recognized as the main regulators of the paracrine mechanism that mediates tissue regeneration [77]. These nanoparticles exert beneficial therapeutic effects similar to those exerted by MSCs and may be potentially powerful tools for cell-free based therapy because they have many advantages over MSCs. Of note, the discovery that MSC exosomes can be used to treat peripheral nerve injury is encouraging. In the peripheral nerve microenvironment, MSC exosomes play an important role in mediating intercellular communication. They can transmit a number of genetic materials, neurotrophic factors and proteins to axons, restoring the homeostasis of the microenvironment, regulating axonal regrowth, and thereby promoting recovery from PNI. These effects are important for adopting MSC exosomes for clinical applications. The therapeutic effect of exosomes reinforces the paradigm that the promotion of tissue regeneration by MSCs is mediated by a paracrine mechanism and provides a new perspective for the development of cell-free therapies [135]. The use of MSC exosomes can eliminate the issues caused by stem cell transplantation. In the future, MSC exosome-based therapy may represent an important field for PNI repair. Although much experimental evidence suggests that the use of MSC exosomes for the treatment of peripheral nerve injury is effective and safe, this field needs to be further researched, and much work is needed to fully determine the potential of MSC exosomes for clinical application.

## Figures and Tables

**Table 1:** The characteristics of exosomes and microvesicles.

	Exosomes	Microvesicles
Size	40-150 nm	100-1000 nm
Size range	narrow	broad
Density	1.18-1.21 g/ml	1.16 g/ml
Origin	The invagination of plasma membrane (PM) forms endosomes; endosomes buds inward to form intraluminal vesicles	The outward shedding of PM
Main formation mechanisms	ESCRT-dependent and independent mechanism	Calcium-dependent mechanism
Maker	CD 9, CD 63, CD 91, TSG 101, ALIX	Integrins, selectins, tissue factors
Content	mRNA, miRNA, lncRNA, DNA, protein, lipid, MHC	mRNA, miRNA, protein

ESCRT: endosomal sorting complex required for transport.

**Table 2:** The beneficial effect of MSCs exosomes for PNI.

Cell source	Exosomal cargo	Effect	The activation of signaling pathway	Reference
hAMSCs	/	Against neuron damage induced by glutamate	the PI3K/Akt signaling pathway	[94]
Gingiva-derived MSCs	/	Promote peripheral nerve regeneration	c-JUN pathway governed repair phenotype of Schwann cells	[95]
rBMMSCs	miR-17-92 cluster	Increase neural plasticity and functional recovery and promote Axonal Growth of Cortical Neurons	the PI3K/protein kinase B/mechanistic target of rapamycin /glycogen synthase kinase 3*β*, or the PTEN/mTOR signaling pathway	[105, 106]
	miR-133b	Improve neural plasticity and functional recovery	/	[107]
rADSCs	PEDF	Ameliorate cerebral I/R injury	Autophagy and apoptotic pathway	[108]
	Multiple factors	Increase neurite outgrowth in vitro and enhance regeneration	/	
Umbilical cord MSCs	let-7b	Alleviate inflammatory reaction by promoting M2 macrophage activation	The signal axis by TLR 4 / NF-*κ*B / STAT-3 / AKT	[122]
rADSCs	miR-30d-5p	Prevent cerebral injury by mediating microglial polarization to M1	Autophagy pathway	[123]
rBMMSCs	/	Enhance neurite remodeling, endogenous angiogenesis, and neurogenesis and reduce inflammation	/	[129, 130]

“/”: not mentioned in the original article; ADSCs: adipose derived mesenchymal stem cells; BMMSCs: bone marrow derived stem cells.
